# The Effect of Season and Breed on Hypothalamic–Pituitary–Adrenal Axis Hormones, Metabolic Hormones, and Oxidative Markers in Ponies and Horses

**DOI:** 10.1111/jvim.70047

**Published:** 2025-03-06

**Authors:** Sarah Alison Vaughn, Margaret B. Lemons, Kelsey A. Hart

**Affiliations:** ^1^ University of Georgia Department of Large Animal Medicine Athens USA

**Keywords:** ACTH, cortisol, insulin, leptin, season

## Abstract

**Background:**

Endocrine function in ponies differs from horses, with seasonally increased concentrations of plasma adrenocorticotropic hormone (ACTH) and an increased risk of insulin dysregulation.

**Hypothesis:**

(1) Season and breed differences exist in concentrations of hypothalamic–pituitary–adrenal axis and metabolic hormones; and (2) systemic oxidative status is significantly different between ponies and horses and correlates with endocrine hormones.

**Animals:**

Thirty‐four healthy Welsh ponies and 14 healthy Quarter horses.

**Methods:**

Blood was collected from Welsh ponies and Quarter horses in the same region during the same weeks in May and October. Concentrations of plasma ACTH, insulin, leptin, derivatives of reactive oxygen metabolites (dROMs), plasma antioxidant capacity (PAC), and serum total cortisol, percent‐free cortisol, and estimated free cortisol concentrations were measured. Linear mixed effects modeling with a random effect for animal was used to determine the effects of season and breed. Correlation coefficients were calculated for relevant variables. Statistical significance was set at *p* < 0.05.

**Results:**

Fall plasma ACTH concentration was significantly higher in ponies compared with horses (*p* < 0.001). Total cortisol concentration was significantly lower in ponies in fall compared with spring (*p* = 0.05; 95% confidence interval [CI] = 0.005–0.934). Insulin concentrations in ponies were significantly higher in fall compared with spring (*p* < 0.001) and compared with horses in fall (*p* < 0.001). In horses, PAC was higher in fall than in spring (*p* = 0.01; 95% CI = −730.2T to −99.26). Correlations varied with season and breed.

**Conclusions:**

Season and breed significantly affect the HPA axis, metabolic hormones, and oxidative status. Our results support breed consideration when interpreting endocrine testing results in horses.

**Abbreviations**
BCS: body condition score
dROM: derivatives of reactive oxygen metabolites
%FC: percent free cortisol
FCC: free cortisol fraction
HPA axis: hypothalamic–pituitary–adrenal axis
ID: insulin dysregulation
PAC: plasma antioxidant capacity
PPID: pituitary pars intermedia dysfunction
ROS: reactive oxygen species

## 1 Introduction

Pituitary pars intermedia dysfunction (PPID) is a common endocrine disorder in horses [1]. A recent systematic review on the epidemiology of PPID found that the most important risk factor for PPID is age and that breed was an equivocal factor related to PPID diagnosis, although lack of breed information and breed‐related statistical analysis in many of the evaluated studies precluded the ability to use breed or type to assess PPID risk [1]. However, some studies have found an increased risk for PPID in certain breeds, particularly ponies and Morgan horses [2, 3, 4, 5, 6, 7, 8, 9]. In a previous survey, 42% of equids diagnosed with PPID were ponies [2], although the proportion of ponies in the equine population likely varies among regions. However, other studies found neither breed nor height correlated with risk level for PPID [10, 11].

The most commonly used diagnostic test for PPID is documentation of increased plasma adrenocorticotropic hormone (ACTH) concentrations [12]. However, several studies have demonstrated that healthy mixed‐breed ponies have increased circulating concentrations of ACTH compared with horses [13, 14], which complicates determining the true prevalence of PPID within and across breeds. Additionally, certain breeds, including Arabian horses, Welsh ponies, and Shetland ponies, experience higher seasonal increases in circulating ACTH concentration during the autumn months even during health, often reaching concentrations above those recommended for PPID diagnosis [15, 16, 17]. Current reference intervals used for the diagnosis of PPID are based on studies that exclude ponies [18], include ponies but do not specify breeds [19], or include ponies but do not specify the breed or number of the study population accounted for by ponies [20, 21].

Breed differences in insulin sensitivity and inflammatory and oxidative responses also vary in equids [22, 23, 24]. Many metabolic measures, including glucose, basal insulin, insulin after an oral sugar test, and leptin concentrations, are moderately to highly heritable in Welsh ponies [25], further suggesting breed may affect endocrine function. Additionally, ex vivo, pony neutrophils produce more reactive oxygen species (ROS) and exhibit lower chemotaxis than horse neutrophils [24]. Differences in oxidative status may be important because neurodegenerative diseases, including PPID, may be associated with increased oxidative stress [26]. No marker of systemic oxidative stress or antioxidant deficiency has been found in horses with PPID [27, 28]. However, evidence in local tissue suggests that decreased antioxidant capacity and accumulation of oxidative stress markers are important in the pathogenesis of PPID [26, 27].

Our objective was to compare hypothalamic–pituitary–adrenal (HPA) axis hormones, metabolic hormones, plasma derivatives of reactive oxygen metabolites (dROM), and plasma antioxidant capacity (PAC) between Quarter horses and Welsh ponies in spring and fall, and determine if these variables vary with season or breed or correlate within breed. We hypothesized (1) that season and breed‐specific differences exist in concentrations of HPA axis and metabolic hormones; and (2) that systemic oxidative status is significantly different between horses and ponies and correlates with endocrine hormones.

## 2 Materials and Methods

### 2.1 Animals

A priori sample size calculations determined that a sample size of 15 Quarter horses and 15 Welsh ponies would provide a power of 90% to detect a mean difference of 50 dROM units, assuming a standard deviation of 35 units, α = 0.05, enrollment of animals on five farms for each breed, and an intra‐cluster correlation of *p* = 0.10 for horses on the same farm. This calculation was based on mean ± SD dROM values in horses (94.9 ± 34.9) and ponies (145.0 ± 30.1) in a pilot study.

Ponies and horses were recruited via social media and personal contacts. The study design was approved by the University of Georgia Clinical Research Committee, and informed client consent was obtained before sampling. To be eligible for inclusion in the study, horses and ponies had to be > 2 years of age, have normal physical examinations at both sample collections, and have a spring plasma ACTH concentration < 50 pg/mL [29]. This plasma ACTH concentration inclusion criterion is based on the Equine Endocrinology Group's 2019 recommendations for diagnosis and treatment of PPID [29] because this study was performed in 2020, and those were the available guidelines when samples were collected and analyzed. Horses and ponies were required to be registered American Quarter horses or Welsh ponies, respectively. Any section Welsh pony was permitted. Animals with current illness, active laminitis, or receiving non‐steroidal anti‐inflammatory medications, levothyroxine, pergolide mesylate, or corticosteroids were excluded. Animals on non‐prescription supplements such as One‐AC (one pony and one horse) and on prescription hydroxyzine (one pony and two horses) were included in the final analysis.

### 2.2 Study Design

Thirty mL of blood was collected via jugular venipuncture into EDTA, sodium heparin, and glass without anticoagulant tubes after assignment of body condition score (BCS) on a scale of 1–9 [30] by one of the study investigators (SV). Although blood collection was timed to occur either before or a minimum of four hours after any grain ration, time‐of‐day sampling and housing varied. All animals were maintained on their home farm, with normal diet, and typical housing and exercise routines during the study period. Sampling was performed on the same animals over a one‐week period in spring (May) and again in fall (October) to assess seasonal changes in HPA axis function [15, 20]. Blood samples were immediately placed on ice upon collection, and serum and plasma were separated within six hours of collection and frozen at −80°C until batch analysis.

### 2.3 Assays

Plasma ACTH and serum total cortisol concentrations were quantified using previously validated chemiluminescent immunoassays [31] within 60 days of collection. Plasma ACTH concentration was quantified on the Immulite 2000 at the Animal Health Diagnostic Center at Cornell University in 2020. Plasma insulin and leptin concentrations and serum total cortisol concentration were measured using a validated radioimmunoassay [32, 33, 34]. Serum free cortisol fraction was determined using a radioactive ultrafiltration ligand‐binding method previously optimized for horses [35] and free cortisol fraction is expressed as percent‐free cortisol (%FC). Free cortisol concentration (FCC) was calculated by multiplying the total cortisol concentration by the free cortisol fraction as previously described [35]. Oxidative markers dROM and PAC were determined in heparinized plasma using a validated photometric system as previously described [36]. Oxidative status assays and free cortisol quantification were completed within 90 days of collection.

### 2.4 Statistical Analysis

All data were assessed for normality using the Shapiro–Wilk test. Not all data were normally distributed, so linear mixed effects modeling with a random effect for animals was used to determine the effects of season and breed on HPA axis hormones, metabolic hormones, and oxidative markers. When applicable, Šídák's multiple comparisons test was used for post hoc analysis. If a significant interaction of season and breed was identified for the variable analyzed, Wilcoxon matched‐pairs signed rank tests were used for within‐breed comparisons (between seasons) or Mann–Whitney tests were used for between‐breed comparisons (within a season). Because ponies came from a variety of farms with varied husbandry and management approaches, the effects of season and farm on HPA axis hormones, metabolic hormones, and oxidative markers were analyzed using linear mixed‐effects modeling with a random effect for pony.

Spearman correlation coefficients were calculated for endocrine and oxidative variables of interest to determine if these variables were significantly associated with either or both breeds in either season. Analysis was performed using commercial statistical software (GraphPad Prism 9, San Diego, CA) with statistical significance set at *p* < 0.05 for all comparisons.

## 3 Results

### 3.1 Animals

Initially, 57 ponies were sampled in spring. Nine of these were unavailable for resampling in fall, and 14 were excluded for not meeting the health criteria. Twelve of these were excluded because they exhibited clinical signs of PPID or other health issues (e.g., hypertrichosis, laminitis, severe heaves), and two were excluded on the basis of ACTH concentrations alone (above the spring cut‐off but without signs of clinical disease). Thirty‐four healthy ponies on five different farms met the inclusion criteria in both seasons and were included in the analysis. Ponies ranged in age from two to 23 years of age (mean, 12.2 ± 6.4 years) with 18 mares, 11 geldings, and five stallions. All were registered, purebred Welsh ponies, with 16 section A (mountain Welsh) ponies, 17 section B (Welsh) ponies, and one section D (Welsh cob) pony. In spring, 29 horses were sampled. Twelve of these were unavailable for resampling in fall, and three were excluded because they were being treated with phenylbutazone during fall sampling. Fourteen healthy Quarter horses on two farms met the inclusion criteria and were included in the analysis. Horses ranged in age from four to 20 years (mean, 11 ± 4.8) with six mares, seven geldings, and one stallion. Although the number of ponies and farms satisfied sample size calculations, there was 1 less horse and fewer farms than needed. Ponies came from a variety of farms: farm 1 (*n* = 6), farm 2 (*n* = 2), farm 3 (*n* = 4), farm 4 (*n* = 4), and farm 5 (*n* = 18). Horses were almost exclusively from the same farm, with a single horse being from one of the Welsh pony locations (farm 1). A Mann–Whitney test was performed comparing the age of the pony group to the age of the horse group, and no significant difference was found between the two groups (*p* = 0.26). Farms were all within a 300‐mile radius in eastern Texas, USA.

### 3.2 Effects of Season and Breed on HPA Axis Hormones

**FIGURE 1:**
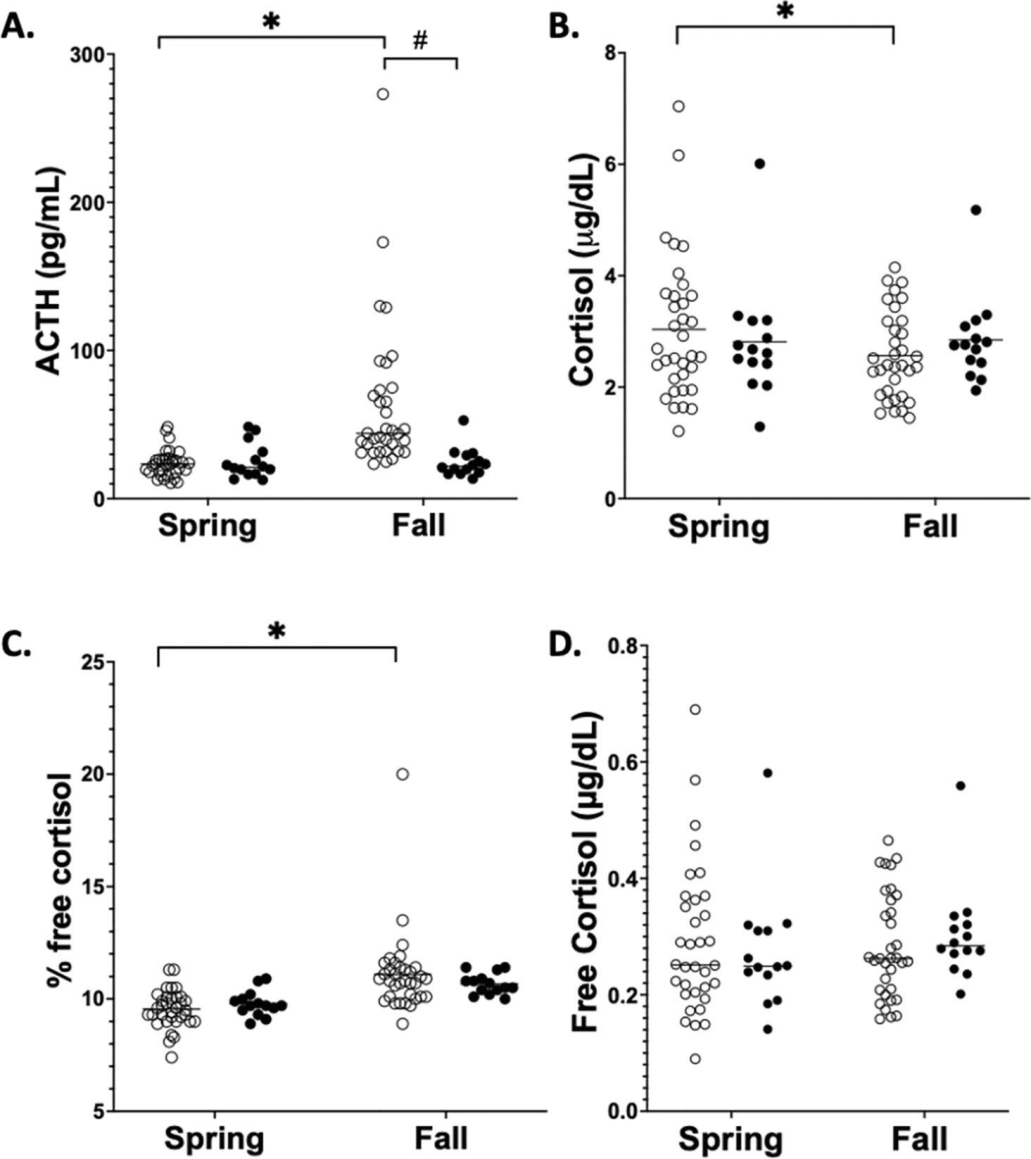
(A) Plasma adrenocorticotropic hormone (ACTH) concentrations, (B) total serum cortisol concentrations, (C) percent free cortisol, and (D) estimated free cortisol concentrations determined in healthy Welsh ponies (*n* = 34) and healthy American Quarter horses (*n* = 14) in both spring (May) and fall (October) in the same year. Each circle represents one animal with ponies shown as open circles and horses as closed black circles. The horizontal lines represent the median for each group. Asterisks denote significant differences within a breed and between seasons. The pound sign denotes significant differences between breeds in the same season. Significance is set as *p* < 0.05.

Spring and fall HPA axis hormone concentrations in ponies and horses and the effects of season and breed on these hormones are shown in Table 1 and Figure 1. Significant effects of both season (*p* = 0.01) and breed (*p* = 0.02) as well as a significant interaction between season and breed (*p* = 0.003) on ACTH concentrations were found. Because of the significant interaction of season and breed on ACTH, within‐breed and within‐season comparisons were examined separately. Ponies had significantly increased ACTH concentrations in the fall compared with the spring (*p* < 0.001), but such was not the case in horses (*p* = 0.93). Plasma ACTH concentrations did not differ significantly between horses and ponies in the spring (*p* = 0.98). In the fall, ACTH concentrations in ponies were significantly increased compared with horses (*p* < 0.001).

**TABLE 1:** *P*‐values and 95% confidence intervals for the effect of season and breed on circulating hypothalamic–pituitary–adrenal axis hormones determined by linear mixed effects modeling with a random effect for the animal.

	*P*	95% CI
**Effect of season on plasma ACTH concentration**	**0.05**	**−32.67 to −5.99**
**Effect of breed on plasma ACTH concentration**	**0.02**	**3.79–33.48**
**Effect of the interaction between season and breed on plasma ACTH concentration**	**0.003**	**−67.62 to −14.24** **14.24–67.62**
Effect of season on serum total cortisol concentration	**0.25**	−0.16—0.59
Effect of breed on serum total cortisol concentration	**0.92**	−0.57—0.51
Effect of the interaction between season and breed on serum total cortisol concentration	**0.18**	−0.25—1.25 −1.25—0.25
**Effect of season on percent free cortisol**	**< 0.001**	**−1.70 to −0.73**
Effect of breed on percent free cortisol	**0.78**	−0.53—0.70
Effect of the interaction between season and breed on percent free cortisol	**0.17**	−1.64—0.31 −0.31—1.64
Effect of season on estimated free cortisol concentration	**0.58**	−0.05—0.03
Effect of breed on estimated free cortisol concentration	**0.95**	−0.06—0.06
Effect of the interaction between season and breed on estimated free cortisol concentration	**0.33**	−0.04—0.11 −0.11—0.04

*Note:* Hormone concentrations were determined in 34 healthy Welsh ponies and 14 Quarter horses in May and October of the same year. Bold text indicates statistical significance (*p* < 0.05).

Four of the 34 ponies (12%) classified as healthy in the spring met the 2019 guidelines for the recommended diagnostic threshold for PPID diagnosis based on resting plasma ACTH concentrations when sampled again during the autumn months (ACTH > 100 pg/mL) [29], despite not having clinical signs in either season. In the spring, these ponies' ACTH concentrations were < 50 pg/mL [29]. These ponies had a mean age of 15 ± 5.3 years. This increase out of the reference range during fall was not seen in any of the horses. An additional 9 of the 34 ponies (26%) that were classified as healthy in the spring, based on an absence of clinical signs of PPID, an ACTH concentration < 50 pg/mL [29], and a mean age of 15.3 ± 6.3 years, had plasma ACTH concentrations in the equivocal zone when sampled during the autumn months (ACTH 50–100 pg/mL) [29]. This finding was true for only one of the 14 horses (7%).

A significant overall effect of season or breed on total cortisol concentrations was not found, and the interaction between season and breed also was not significant. Total cortisol concentrations were significantly increased in ponies in the spring compared with the fall (*p* = 0.05; 95% CI = 0.005–0.934). Also, no significant effect of breed on %FC was observed, but the effect of season on %FC was significant (*p* < 0.001). The interaction between season and breed was not significant. Percent free cortisol in ponies was significantly increased in the fall compared with the spring (*p* < 0.001; 95% CI = −2.15 – −0.943). No significant effect of season or breed on FCCs was found, and the interaction between season and breed also was not significant.

### 3.3 Effects of Season and Breed on Metabolic Hormones and Body Condition Score

**FIGURE 2:**
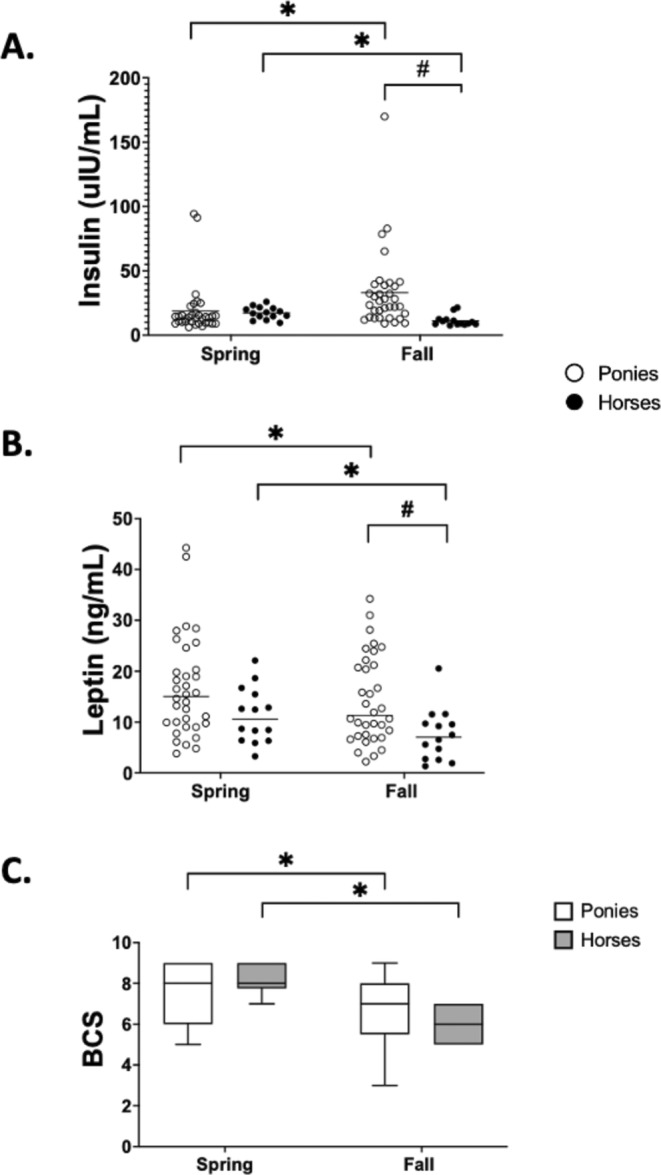
Plasma insulin concentrations, (B) plasma leptin concentrations, and (C) body condition score (BCS) determined in healthy Welsh ponies (*n* = 34) and healthy American Quarter horses (*n* = 14) in both spring (May) and fall (October) in the same year. The horizontal lines represent the median for each group (A, B). Each circle represents one animal with ponies shown as open circles and horses as closed black circles (A, B). Open boxes represent ponies and gray boxes represent horses (C). Asterisks denote significant differences within a breed and between seasons. The pound sign denotes significant differences between breeds in the same season. Significance is set as *p* < 0.05.

Spring and fall metabolic hormone concentrations and BCS in ponies and horses and the effects of season and breed on these variables are shown in Table 2 and Figure 2. No significant effect of season or breed alone on insulin concentrations was found, but a significant interaction between season and breed (*p* = 0.01) was observed. Because of the significant interaction of season and breed with insulin, within‐breed and within‐season comparisons were examined separately. Insulin concentrations in ponies were significantly increased in the fall compared with the spring (*p* < 0.001) whereas the opposite season pattern was observed in horses, with insulin concentrations increased in the spring compared with the fall (*p* < 0.001). Insulin concentrations did not differ significantly between horses and ponies in the spring (*p* = 0.07). In the fall, insulin concentrations in ponies were significantly higher than in horses (*p* < 0.001).

**TABLE 2:** *P*‐values and 95% confidence intervals for the effect of season and breed on circulating metabolic hormones and body condition score (BCS) determined by linear mixed effects modeling with a random effect for animal.

	P	95% CI
Effect of season on insulin concentration	0.28	−11.75—3.49
Effect of breed on insulin concentration	0.05	−0.03—23.17
**Effect of the interaction between season and breed on insulin concentration**	**0.01**	**−35.38 to −4.91** **4.91–35.38**
**Effect of season on leptin concentration**	**< 0.001**	**1.50–4.71**
**Effect of breed on leptin concentration**	**0.02**	**1.03–11.18**
Effect of the interaction between season and breed on leptin concentration	0.39	−4.58—1.82 −1.82—4.58
**Effect of season on BCS**	**< 0.001**	**1.23–2.02**
Effect of breed on BCS	0.93	−0.79—0.73
**Effect of the interaction between season and breed on BCS**	**< 0.001**	**−2.25 to −0.67** **0.67–2.25**

*Note:* Hormone concentrations were determined in 34 healthy Welsh ponies and 14 Quarter horses in May and October of the same year. Bold text indicates statistical significance (*p* < 0.05).

A significant effect of both season (*p* < 0.001) and breed (*p* = 0.019) on leptin concentrations was found, but the interaction between season and breed was not significant. Leptin concentrations in ponies and horses were significantly increased in the spring compared with the fall (*p* = 0.01; 95% CI = 0.428–4.40 and *p* = 0.01; 95% CI = 0.699–6.89, respectively). Leptin concentrations differed significantly between ponies and horses in the fall (*p* = 0.02; 95% CI = 0.787–12.81) but not in the spring (*p* = 0.09; 95% CI = −0.593—11.43).

A significant effect of season (*p* < 0.001) but not breed on BCS was found. The interaction between season and breed also had a significant effect on BCS (*p* < 0.001), and thus within‐breed and within‐season comparisons were examined separately. Body condition scores in ponies and horses were both significantly lower in the fall than in the spring when compared within breeds (*p* < 0.001). Body condition score was not significantly different between breeds in either season.

### 3.4 Effects of Season and Breed on Oxidative Markers

**FIGURE 3:**
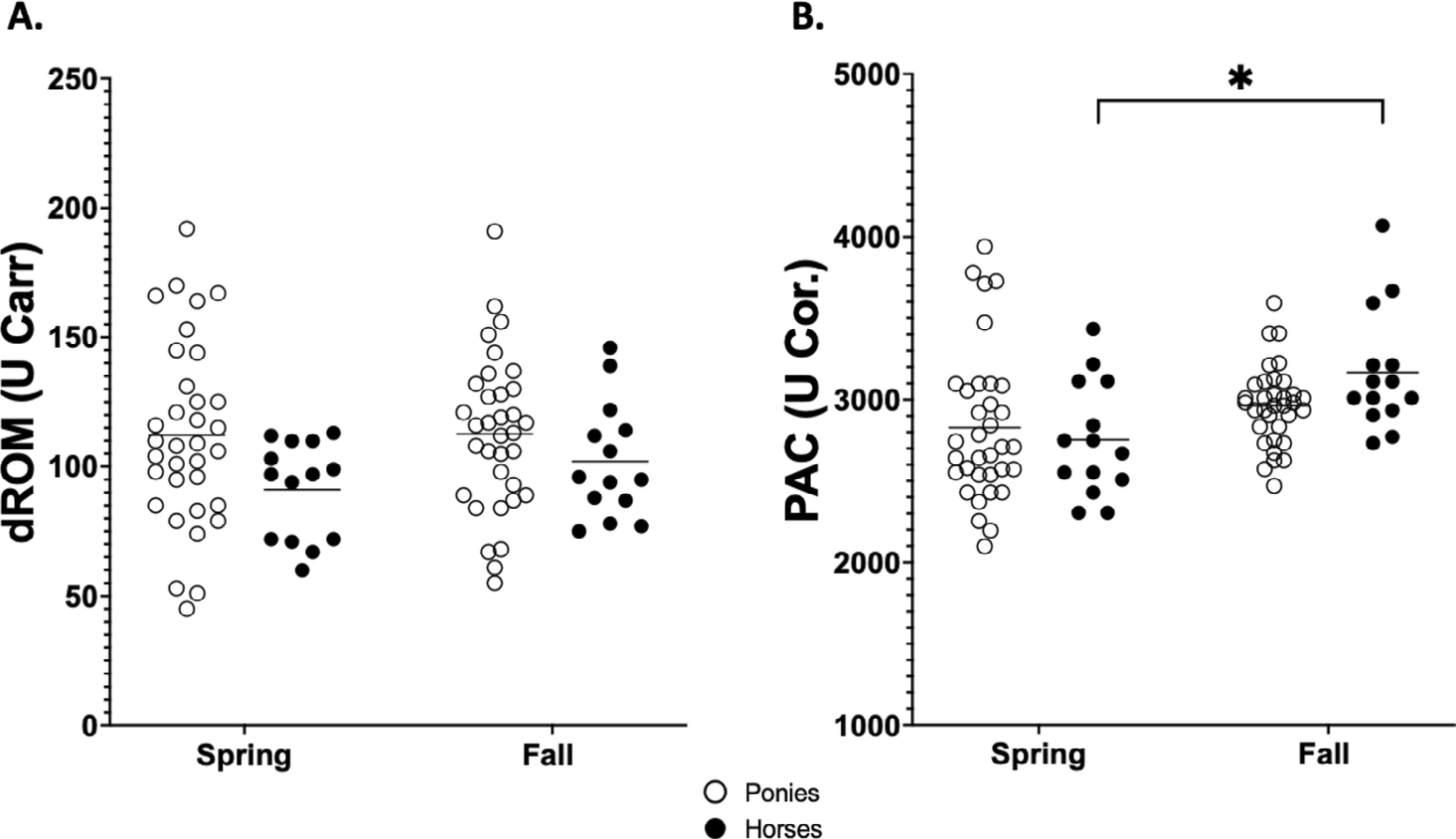
Plasma derivatives of reactive oxygen metabolites (dROM), and (B) plasma antioxidant capacity (PAC) determined in healthy Welsh ponies (*n* = 34) and healthy American Quarter horses (*n* = 14) in both spring (May) and fall (October) in the same year. Each circle represents one animal with ponies shown as open circles and horses as closed black circles. The horizontal lines represent the median for each group. Asterisks denote significant differences within a breed and between seasons. Significance is set as *p* < 0.05.

Spring and fall concentrations of oxidative markers in ponies and horses and the effects of season and breed on these variables are shown in Table 3 and Figure 3. A significant effect of breed or season on dROM was not observed, and the interaction between season and breed was not significant. A significant effect of season on PAC (*p* = 0.001) was found, but not a significant effect of breed or interaction between season and breed. Plasma antioxidant capacity was significantly increased in horses in the fall when compared with the spring (*p* = 0.01; 95% CI = −730.2 to −99.26).

**TABLE 3:** *P*‐values and 95% confidence intervals for the effect of season and breed on plasma oxidative markers determined by linear mixed effects modeling with a random effect for animal.

	*P*	95% CI
Effect of season on dROM	**0.19**	−14.22—2.95
Effect of breed on dROM	0.07	−1.33—32.87
Effect of the interaction between season and breed on dROM	0.23	−6.72—27.61 −27.61—6.72
**Effect of season on PAC**	**0.001**	−438.0 to −111.6
Effect of breed on PAC	0.44	−233.1—103.4
Effect of the interaction between season and breed on PAC	0.09	−46.50—606.2 −606.2—46.50

*Note:* Concentrations of derivatives of reactive oxygen metabolites (dROM) and plasma antioxidant capacity (PAC) were determined in 34 healthy Welsh ponies and 14 Quarter horses in May and October of the same year. Bold text indicates statistical significance (*p* < 0.05).

### 3.5 Effects of Farm on Metabolic and Oxidative Variables in Ponies

No significant effect of farm or interaction between season and farm for insulin or leptin concentrations or on dROM was found. Although no significant effect of the farm on BCS was observed, the interaction between season and farm was significant (*p* = 0.04). Farm had a significant effect on PAC (*p* < 0.001), but the interaction between season and farm was not significant.

### 3.6 Correlations Between Hormones and Oxidative Markers

Significant correlations between selected endocrine and oxidative variables are shown in Table 4. Derivatives of reactive oxygen metabolites were significantly correlated with several other markers. Specifically, dROM were negatively correlated with age in ponies in the spring and positively correlated with age in horses in the spring, positively correlated with cortisol concentrations in ponies in the spring, positively correlated with leptin concentrations in ponies in the fall, and in horses in both seasons, positively correlated with BCS in both ponies and horses in the fall, and positively correlated with PAC in ponies in the spring. Plasma antioxidant capacity was not significantly correlated with any hormones or body condition, but was positively correlated with age in ponies in the spring.

**TABLE 4:** *P*‐values, *r* values, and 95% confidence intervals for the Spearman correlation coefficients calculated for endocrine, clinical, and oxidative parameters in 34 healthy Welsh ponies and 14 Quarter horses in May and October of the same year.

	Ponies						Horses					
	Spring			Fall			Spring			Fall		
	P	*r*	95% CI	P	*r*	95% CI	P	*r*	95% CI	P	*r*	95% CI
**dROM & age**	**0.01**	**−0.433**	**−0.68 to −0.10**	0.10	−0.286	−0.58—0.07	**0.02**	**0.645**	**0.13—0.89**	0.16	0.415	−0.19—0.79
**dROM & cortisol**	**0.05**	**0.345**	**−0.002—0.62**	0.34	0.168	−0.19—0.49	0.65	0.135	−0.44—0.63	0.39	0.248	−0.34—0.70
**dROM & leptin**	0.23	0.211	−0.15—0.52	**0.02**	**0.390**	**0.05—0.65**	**0.03**	**0.598**	**0.08—0.86**	**0.05**	**0.547**	**0.01—0.84**
**dROM & BCS**	0.92	−0.018	−0.37—0.34	**0.04**	**0.360**	**0.01—0.63**	0.11	0.442	−0.13—0.79	**0.04**	**0.572**	**0.04—0.85**
**dROM & PAC**	**0.02**	**0.393**	**0.05—0.65**	0.13	0.266	−0.09—0.56	0.25	0.330	−0.26—0.74	0.60	−0.153	−0.64—0.43
**PAC & age**	**0.03**	**−0.381**	**−0.64 to −0.04**	0.24	−0.209	−0.52—0.14	0.46	0.225	−0.39—0.70	0.64	0.143	−0.46—0.65
**ACTH & age**	0.69	0.072	−0.28—0.41	**0.03**	**0.377**	**0.03—0.64**	**0.02**	**0.655**	**0.14—0.90**	**0.03**	**0.619**	**0.09—0.88**
**ACTH & cortisol**	**0.01**	**0.429**	**0.10—0.68**	0.41	−0.146	−0.47—0.21	0.84	−0.059	−0.58—0.50	0.16	0.402	−0.18—0.78
**Insulin & cortisol**	0.71	0.067	−0.29—0.41	0.50	0.119	−0.24—0.45	**0.02**	**0.645**	**0.16—0.88**	0.19	0.371	−0.21—0.76
**Insulin & BCS**	0.08	0.309	−0.05—0.60	**0.01**	**0.427**	**0.09—0.68**	0.96	−0.015	−0.55—0.53	0.05	0.534	−0.01—0.84
**Insulin & leptin**	0.12	0.271	−0.0 to −0.57	**0.03**	**0.372**	**0.03—0.64**	0.09	0.477	−0.09—0.44	0.30	0.297	−0.29—0.72
**Leptin & BCS**	**< 0.001**	**0.603**	**0.32—0.79**	**< 0.001**	**0.642**	**0.37—0.81**	0.28	0.311	−0.28—0.73	0.05	0.539	−0.01—0.84

*Note:* Correlations that were not significant in either breed or season are not shown. Bold text indicates statistical significance (*p* < 0.05).

Abbreviations: ACTH = adrenocorticotropic hormone; BCS = body condition score; dROM = derivatives of reactive oxygen metabolites, cortisol refers to total serum cortisol; PAC = plasma antioxidant capacity.

Plasma ACTH concentration was moderately to strongly correlated with age in horses in both seasons, but in ponies, age and ACTH were moderately positively correlated only in the fall. Plasma ACTH concentration was also weakly positively correlated with total cortisol concentration in ponies in the spring. Cortisol was strongly positively correlated with insulin in horses in the fall, but not in the spring, or in either season in ponies. Insulin was positively correlated with BCS in ponies in the fall, but not in the spring. Insulin was also positively correlated with leptin in ponies in the fall. Lastly, leptin was strongly positively correlated with BCS in ponies in both seasons, but not in horses.

## 4 Discussion

These data support our first hypothesis that season and breed‐specific differences exist in concentrations of HPA axis and metabolic hormones and that seasonal changes in these hormones are more extreme in Welsh ponies than in Quarter horses. These data indicate that season or breed or both significantly impact ACTH, insulin, and leptin concentrations, %FC, as well as BCS. Other studies evaluating breed differences in ACTH concentrations had conflicting results, with some finding ACTH concentrations increased in ponies compared with horses without considering seasonal differences [5, 14], some finding that ponies have significantly increased ACTH concentrations compared with horses in fall [13, 15, 16], and some finding no difference [37, 38]. However, many of these studies used mixed‐breed ponies or did not provide information on breed. These studies are further confounded by evidence that in addition to season [15, 20, 21] and possibly breed, ACTH is also affected by factors such as geography [19, 39], stress [40], and age, independent of disease [41]. In contrast, the ponies and horses in our study were all in the same geographic region, all had normal physical examinations and were apparently healthy, were all purebred registered animals without breed variation, and no significant difference in age existed between pony and horse groups (*p* = 0.28).

Our findings also provide partial support for our second hypothesis, that systemic oxidative status is significantly different between Welsh ponies and Quarter horses, and that markers of oxidative status correlate with HPA axis and metabolic hormones. Our work also demonstrated that season and breed do not have a significant effect on dROM, but may significantly impact PAC, and that relationships between these variables vary between seasons and breeds. However, although the effect of farm was assessed in ponies, it could not be assessed in the horse group. Additionally, dietary antioxidant content was not assessed in our study, and thus it is possible that the identified seasonal and breed effects on PAC may reflect dietary differences rather than inherent breed differences.

In our study, 38% of the ponies classified as healthy in spring (ACTH concentrations < 50 pg/mL) [29] were either in the equivocal diagnostic (ACTH 50–100 pg/mL) [29] or positive (ACTH > 100 pg/mL) [29] category for PPID based on resting ACTH concentrations when sampled again in the fall despite not having clinical signs. Although an equivocal ACTH concentration without clinical signs of PPID should be interpreted as a negative result, these results emphasize the breed differences in seasonal increases that must be considered when interpreting these results. Additionally, 12% of the ponies had ACTH concentrations that fell in the PPID‐positive range. It is possible that this finding is a consequence of being truly positive for PPID with early‐stage disease. However, given the lack of any clinical signs and the young age of several of these animals, it is also possible that the current diagnostic criteria do not account for the magnitude of the increase that Welsh ponies experience in plasma ACTH concentrations in the autumn. A Welsh pony being tested for non‐specific clinical signs, such as tendon or ligament injury or chronic skin disease, could be misdiagnosed as being positive for PPID if tested in the fall with equivocal or positive results. This observation lends support to recent calls for the consideration of breed when interpreting diagnostic testing results for PPID, particularly in ponies [15, 16].

Somewhat unexpected was the finding that total cortisol concentrations in ponies were significantly lower in the fall compared with the spring, despite the increase in ACTH concentrations. However, although total cortisol concentrations were significantly decreased in ponies in the fall compared with the spring, %FC was significantly increased in the fall along with ACTH concentrations. One other study observed a dyssynchronous relationship between ACTH and cortisol concentrations in horses in the fall [42]. Because FCCs were not different between seasons, less cortisol is circulating overall, but the same absolute amount of free cortisol remains unbound, leading to an increase in the free percentage. This observation suggests altered stimulation of, or response from, the adrenal glands. It is possible that fall ACTH concentrations in ponies are falsely increased by increased concentrations of cross‐reactive hormones [43, 44], or that ACTH produced by Welsh ponies during the fall is less bioactive. Decreased seasonal adrenal responsiveness to exogenous ACTH administration has been reported in the fall compared with the spring in other species, such as goats [45], suggesting that another possibility could be a seasonal resistance to ACTH that occurs at the level of the adrenal glands.

Although it is widely accepted that differences in insulin sensitivity exist between ponies and horses [22, 23], our study supports previous work suggesting that the interaction of season and breed has a significant effect on insulin concentrations [17]. Insulin concentrations in ponies were significantly higher in the fall compared with the spring, whereas the opposite seasonal pattern was observed in horses. Although obese equids have been found to have increased insulin concentrations compared with lean controls [33], the Welsh ponies in our study had significantly lower BCS in the fall compared with spring, and no significant difference was found between pony and horse BCS in the fall. Thus, the increase in insulin concentrations that ponies experienced in the fall was not associated with increased BCS. Additionally, serum total cortisol concentrations were lower in Welsh ponies in the fall compared with spring. Although %FC in ponies was significantly higher in the fall compared with spring, as described above, this increase was paired with a decrease in serum total cortisol concentration and an unchanged FCC, indicating that there is a similar absolute amount of free cortisol circulating. Therefore, it is unlikely that the higher insulin concentrations in the fall were caused by antagonism from endogenous glucocorticoids.

Reactive oxygen species, measured as dROM [46], come from a number of sources, including leukocytes and cellular glucose metabolism, and can be produced both in health and in response to endogenous or exogenous stress. Because dROM are also involved in a number of cellular functions, cellular damage occurs when insufficient antioxidants are available to balance the dROM and oxidative stress that occurs. In other species, an association exists among obesity, insulin dysregulation, and increased oxidative stress [47]. In equids, various correlations exist among circulating inflammatory cytokines, equine metabolic syndrome (EMS), obesity, and insulin concentrations [48, 49, 50], with ponies at increased risk for insulin dysregulation (ID) as discussed above [22, 23]. Additionally, neutrophils isolated from ponies produce more ROS than those isolated from horses [24]. Thus, it was unexpected that we did not observe any significant differences in dROM within or among breeds. Additionally, in Welsh ponies, a wider range of dROM was found than in horses in both spring and fall, with the upper range being more extreme than what was observed in the Quarter horses. These data suggest that some Welsh ponies may experience more oxidative stress than Quarter horses, but that this finding is not uniform throughout the breed. It is also possible, because of this increased variability in comparison to what was expected for our sample size calculations, and the fact that we did not have the desired number of horses, that our study was underpowered to detect significant differences in dROM among breeds.

Although dROM did not differ significantly between breeds or seasons in this population, they did correlate with multiple endocrine variables. One surprising finding was that dROM were negatively correlated with age in ponies in both seasons. Aging is associated with chronic inflammation and oxidative stress [9, 28], and a positive correlation between dROM and age would be expected, such as that seen in horses in spring. Evidence exists that ACTH and the related cleavage product α‐melanocyte stimulating hormone, both of which are increased in PPID, are strongly anti‐inflammatory, [9, 51, 52] and it is possible that the high circulating concentration of ACTH seen in ponies in fall counteracts the more inflammatory hormones also increased in fall (e.g., insulin) [53]. This hypothesis would not, however, explain the same pattern observed in spring, and thus it is likely that additional differences exist between horses and ponies in inflammatory responses or management of oxidative balance that warrant further study.

The only difference in PAC that can balance any increases in dROM was that horses had increased PAC in fall compared with spring. This finding may be related to differences in diet between spring and fall. Although none of the animals changed feed between seasons, antioxidant content in available grass and hay can vary substantially. Although no significant correlations were found between PAC and individual endocrine hormones, the multiple differences in hormone concentrations observed between Welsh ponies and Quarter horses in the fall may be interrelated. One explanation is that ponies experience increased concentrations of dROM and need to utilize a larger proportion of PAC to neutralize these. Additionally, we measured systemic markers of oxidative status. Also, if breed differences in oxidative status and antioxidant pathways exist, they may occur locally at the tissue level rather than systemically, much like in PPID [26, 27, 28].

Our study had some limitations. Despite initially sampling almost twice as many horses as needed to meet sample size calculations, our Quarter horse group still fell short of our target population number because of a large number of horses not being available for the second sample collection. Consequently, our study likely was underpowered to detect smaller differences both between breeds and within the Quarter horses between seasons. Additionally, whereas the Welsh ponies were spread across five farms, allowing the effect of farm to be examined, all but one horse came from a single farm. This factor also contributed to our study being underpowered and meant that the effect of farm could not be considered in the Quarter horse group. Management factors, such as diet and exercise, can affect a number of the measured variables, and given that these factors inevitably differ from farm to farm, the inability to investigate farm effects in horses was a substantial limitation. Another limitation was the exclusion of animals with increased ACTH concentrations in our definition of healthy for our inclusion criteria. If healthy ponies do have increased concentrations of ACTH, they may have been unnecessarily excluded. However, only two ponies were excluded on the basis of spring ACTH concentrations alone without any clinical signs of PPID.

Additionally, our samples were not all taken at the same time of day, with samples being drawn from early morning through late afternoon. Cortisol concentrations in horses may follow a circadian rhythm, but they have been shown to be easily disrupted or inconsistent [54, 55, 56]. Another study found that plasma ACTH concentration varies considerably both within and among individual horses and does not follow a well‐defined rhythm [42].

Finally, although insulin does not have a circadian rhythm in horses [57], it is affected by the fed state. In our study, ponies and horses were not tested within four hours of a grain meal. However, given the variability in housing, with some animals in stalls with hay, others on lush pastures, and some on dry lots, as well as the variability of time since the last grain meal, ranging from 5 to > 12 h, we chose not to exclude animals based on insulin concentrations. In the spring, two ponies met the Equine Endocrine Group's 2020 diagnostic criteria for ID, with insulin concentrations > 50 μ IU/mL [58], and were included in the analysis. In the fall, that number increased to five ponies. No horses met this criterion regardless of season. Interestingly, the two ponies that had high insulin concentrations in the spring both experienced a seasonal decrease in insulin concentration in the fall, although they still met the diagnostic threshold for ID, which means they did not contribute to the significant increase in insulin concentrations seen in Welsh ponies in the fall. Although we did assess generalized adiposity to help assess metabolic status, we did not measure a cresty neck score, which can be an additional predictor of ID in equids [59] and would have been a useful tool for interpreting some of these seasonal changes in the pony population.

Our findings suggest significant differences in HPA axis hormones, metabolic hormones, and PAC exist between horses and ponies, and that these differences are affected by season. Furthermore, although dROM correlate with many of these hormones, the correlations are season and breed‐specific. Our work further supports the need to take breed into consideration when interpreting basal plasma ACTH concentrations to diagnose PPID. Further study is needed to determine if or how local and systemic oxidant responses impact endocrine disease risk within and among breeds.

## Disclosure

Authors declare no off‐label use of antimicrobials.

## Ethics Statement

Institutional Animal Care and Use Committee approval was granted for this study by University of Georgia College of Veterinary Medicine Clinical Research Committee and informed client consent was obtained before sampling. Authors declare human ethics approval was not needed.

## Conflicts of Interest

Dr. Kelsey Hart has served as an unpaid consultant for and had travel expenses covered by Boehringer Ingelheim, producer of a Food and Drug Administration (FDA) labeled treatment for pituitary pars intermedia dysfunction in horses. She was not involved in the selection of animals for sampling. Classification of animals was decided using the published consensus statement's recommended cut‐off values. The other authors declare no conflicts of interest.
